# Screening of an Endophyte Transforming Polydatin to Resveratrol from *Reynoutria Japonica* Houtt and the Optimization of Its Transformation Parameters

**DOI:** 10.3390/molecules25204830

**Published:** 2020-10-20

**Authors:** Jin Liu, Xueqing Zhang, Ting Yan, Faling Wang, Jing Li, Lingyun Jia, Jingming Jia, Gaosheng Hu

**Affiliations:** 1 School of Traditional Chinese Materia Medica, Shenyang Pharmaceutical University, Shenyang 110016, China; 15733239775@163.com (J.L.); zhangxq383@163.com (X.Z.); yantinglingxiao@163.com (T.Y.); wangfaling227@163.com (F.W.); jingli0306@163.com (J.L.); jialingyun2003@126.com (L.J.); jiajingming@163.com (J.J.); 2 China-Korea Joint Laboratory of Molecular Pharmacognosy, Shenyang Pharmaceutical University, Shenyang 110016, China

**Keywords:** resveratrol, polydatin, microbial biotransformation, *Bacillus aryabhattai*, optimization, *Reynoutria japonica*

## Abstract

Resveratrol showed various kinds of bioactivities, such as antioxidant, antimicrobial, anticancer effects and, therefore, has been used widely as an important ingredient in medication, healthy foods and cosmetics. However, in nature, resveratrol usually exists at low content and more often exists as polydatin. Therefore, it becomes important to find the cost-effective and environmental-friendly way to transform polydatin to resveratrol. In this study, endophytes were isolated from the rhizome tissue of *Reynoutria japonica* and screened for transforming polydatin to resveratrol using reversed-phase high-performance liquid chromatography (RP-HPLC) and confirmed by liquid chromatography-mass spectrometry (LC-MS) and nuclear magnetic resonance (NMR) spectroscopy. A bacterium identified as *Bacillus aryabhattai* using 16S rRNA phylogenetic tree analysis showed highest transformation rate. The transforming conditions were optimized including substrate concentration, substrate addition time, culture temperature and inoculation ratio. Our results demonstrated that the bacteria isolated from *R. japonica* rhizome tissue showed high activity in transforming polydatin into resveratrol. Crude extract of *R. japonica* root and rhizome (RJE) was also tested as substrate and it was found that the transformation was significantly inhibited at 10.0 mg/mL RJE. Emodin at equivalent concentration of 10.0 mg/mL RJE showed no inhibition activity, and glucose content in RJE was trace and far from enough to exhibit the inhibitory activity. Successive solvent partition followed by an inhibition activity assay revealed that the ethyl acetate fraction showed the main inhibition activity. However, due to the coexistence of polydatin and compounds with inhibitory activity, the concentration of RJE can only be used at limited concentration as substrate.

## 1. Introduction

3,4′,5-trihydroxy-1,2-diphenylethylene, a non-flavonoid polyphenolic compound, is well known as resveratrol. It has been found in the fruit skin and seed of grape fruit (lower than 2 μg/g), mulberry (about 94.5 μg/g), root tissue of *Arachis hypogaea* (peanut) (38.91 μg/g), and *Reynoutria japonica* Houtt root (670–3420 μg/g dry weight) [1,2]. It has been reported that resveratrol showed significant clinical effects, such as anti-cancer and anti-oxidant [3,4,5,6,7,8,9,10,11]. As an application in medication, functional foods and cosmetics development, the market demand for resveratrol is increasing rapidly.

Currently, there are several methods for producing resveratrol including solvent extraction, chemical synthesis, biosynthesis and biotransformation [12,13,14]. Due to the low contents of resveratrol in plant materials, the efficiency of the solvent extraction method is low and cost-ineffective [15]. A chemical synthesis method improved the yield rate but with environmental unfriendly solvent and the synthesis procedure is complicated [16]. Kevin et al. reported a strategy over expressing a 4-coumaroyl-CoA ligase (*4CL*) and stibene synthase (*STS*) to produce resveratrol in engineered *Escherichia coli* cells and the final yield reached 100 mg/L resveratrol, which is still to be improved to reach industrial scale [17,18]. As for the biotransformation method, it showed significant advantages in operation simplicity, environment-friendly and mild conditions [18], which is becoming increasingly accepted. There are mainly two transformation methods using crude enzyme or purified enzymes, and using microbes including bacteria and fungi. It has been reported that polydatin has been successfully transformed into resveratrol by several fungi including *Rhizopus microsporus* [19], *Aspergillus niger* [20,21], *A. oryzae* [22], *Bacillu fafensis* [23] and genetically engineered yeast [24,25].

*Reynoutria japonica* Houtt, a Polygonaceae perennial plant, is well known for its strong vitality. Besides, it is also the main resource of resveratrol (C_14_H_12_O_3_) 0.67–3.42 mg/g and polydatin (C_20_H_22_O_8_) 10–20 mg/g [1,2] in the plant kingdom. We determined the contents of polydatin, resveratrol and emodin in different tissues of *R. japonica*., and found the contents of three compounds in the rhizome and root phloem were significantly higher than that in xylem tissue. These compounds especially resveratrol was believed to be phytoalexin against microbe pathogen. Plant endophytes are specific microbes that can live inside the plant tissue without severe pathogenicity. The specific living environment made the endophytes more attractive and selective in microbe mediated biotransformation [26,27]. In this study, endophytes were isolated from fresh phloem tissue of *R. japonica* and screened for polydatin hydrolysis to produce resveratrol, in order to provide an effective way to produce resveratrol using a biotransformation method.

## 2. Results

### 2.1. Contents Determination of Polydatin, Resveratrol and Emodin in Different Tissues of R. japonica

As shown in Table 1, the contents of polydatin, resveratrol and emodin in phloem tissue of the rhizome and root were several times higher than that in xylem tissue. In leaf and stem tissue, the contents of three compounds are very low and even hard to detect. Secondary metabolites are usually phytoalexins produced for self-defense under stress conditions. A similar distribution was found in several medicinal plants [28,29]. From this point of view, the phloem contained a higher level of defense compounds for the purpose of self-defense in soil and mainly against microbes. Therefore, we chose phloem tissue for the endophyte isolation and activity screening.

### 2.2. Screening of Resveratrol Producing Endophytes

**Figure 1:**
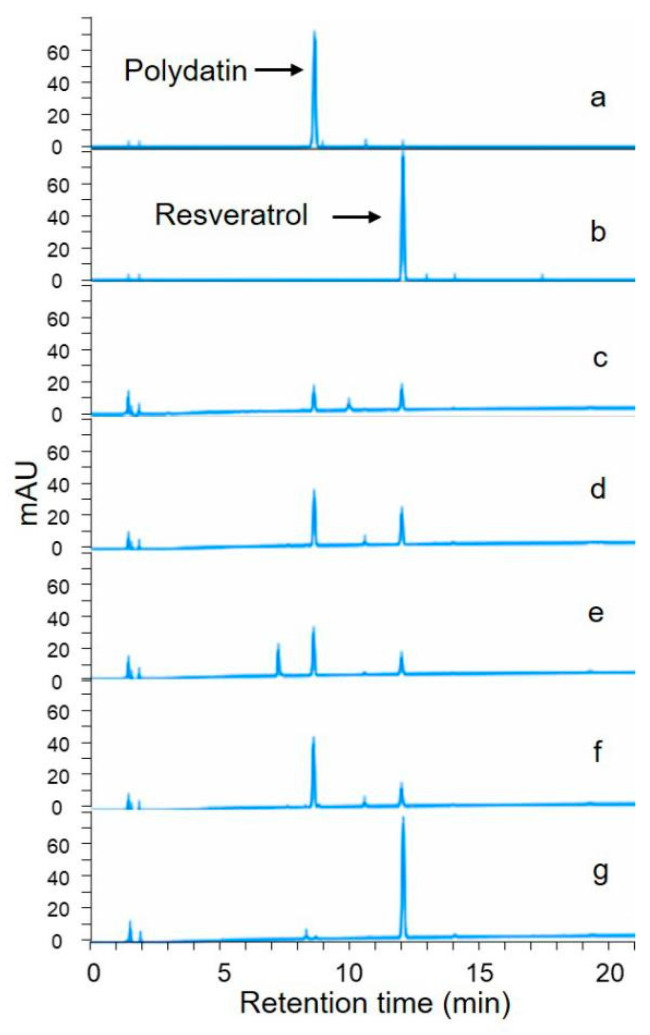
Representative high-performance liquid chromatography (HPLC) chromatogram of biotransformation activity screening of positive strains. (**a**): polydatin standard; (**b**): resveratrol standard; (**c**): Z1; (**d**): Z3; (**e**): Z4; (**f**): Z5; (**g**): X1.

In total, five fungi (Z1–Z5) and six bacterium strains (X1–X6) were isolated from rhizome phloem tissue. Screening results showed, that (Figure 1) four fungi and one bacteria strain showed polydatin biotransformation activity and X1 showed highest activity under current conditions (Table 2). In the rest of this study, X1 was characterized and used for biotransformation optimization.

### 2.3. Strain Identification of X1

**Figure 2:**
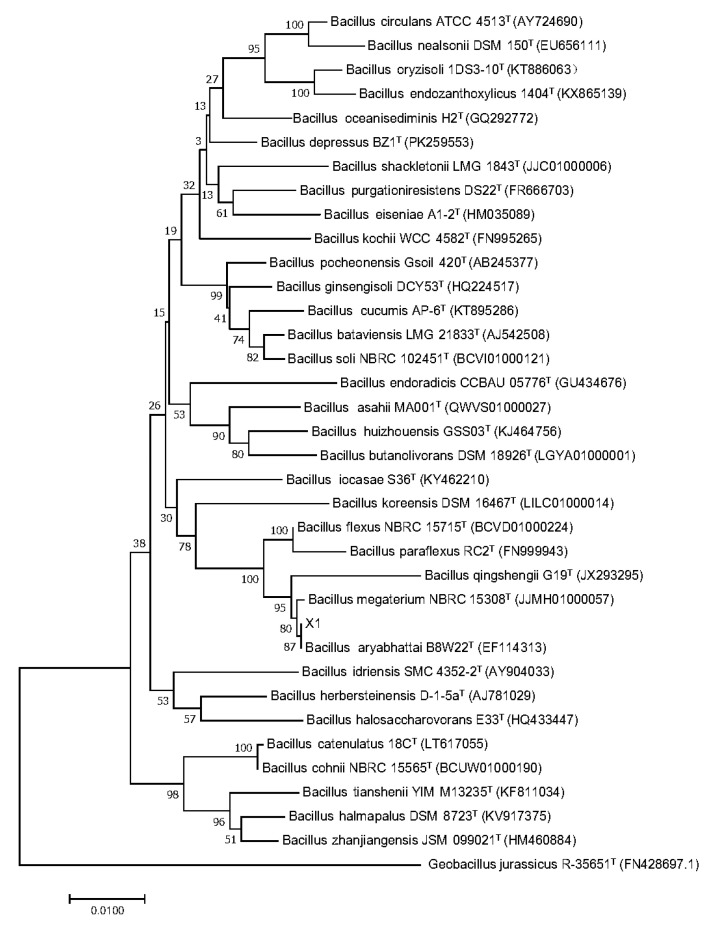
Phylogenetic tree of X1 16S rRNA with other *Bacillus* strains.

We amplified and sequenced 16S rRNA fragments of the X1 strain. A blast n search result in EzBiocloud server indicated similarity of over 99% with several bacillus strains. Phylogenetic tree revealed, that (Figure 2) it has the highest homology with *Bacillus aryabhattai* B8W22^T^ with 99.79% similarity.

### 2.4. Identification of Biotransformation Product Using Liquid Chromatography-Mass Spectrometry (LC-MS) and Nuclear Magnetic Resonance (^1^H-NMR)

**Figure 3:**
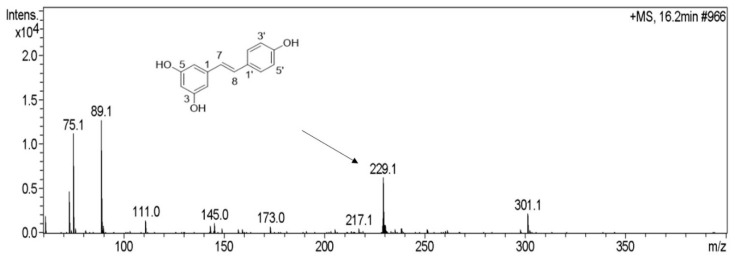
Mass spectrum of biotransformation product under positive ionization mode.

As shown in Figure 3, under positive ionization mode, an ion with 229.1 was detected from the biotransformation product of X1, which was in accordance with the [M + H]^+^ mass data of resveratrol.

**Figure 4:**
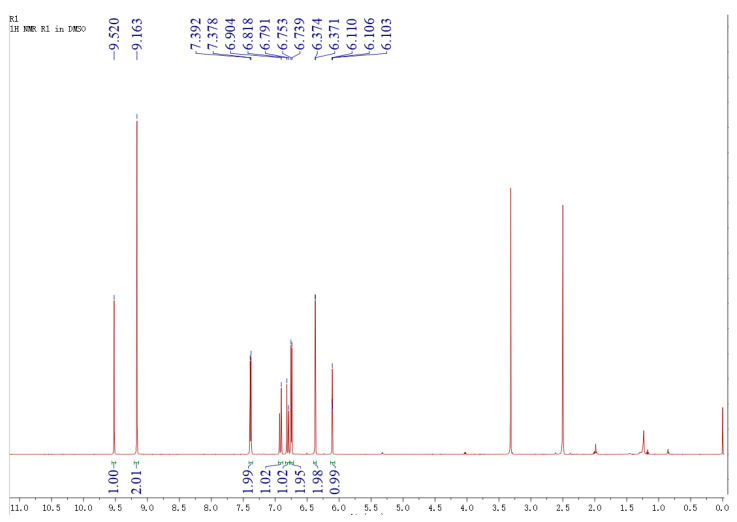
Nuclear magnetic resonance (^1^H-NMR) spectrum of purified resveratrol by X1.

To determine the stereoisomerism of transformation product by X1, the target product was purified using a preparative high-performance liquid chromatography (HPLC) and analyzed using nuclear magnetic resonance (^1^H-NMR) as shown in Figure 4. On the basis of the ^1^H-NMR spectroscopic data (Figure 4), Two double bond proton signals at δ H 6.92 (1H, d, *J* = 16.2 Hz) and δ H 6.80 (1H, d, *J* = 16.2 Hz) were observed, which were deduced to be a pair of trans double bonds. Meanwhile, seven aromatic proton signals at δ H 7. 39 (2H, d, *J* = 8.4 Hz) and δH 6. 75 (2H, d, *J* = 8.4 Hz) indicated an AA’XX’ coupled system in a sub-structure. The aforementioned spectroscopic data suggested that the construction was similar or identical to trans-3,4′ trihydroxistilbenne [29].

### 2.5. Optimization of Biotransformation Conditions of X1 Strain

#### 2.5.1. Plackett–Burman (PB) Test Result

**Figure 5:**
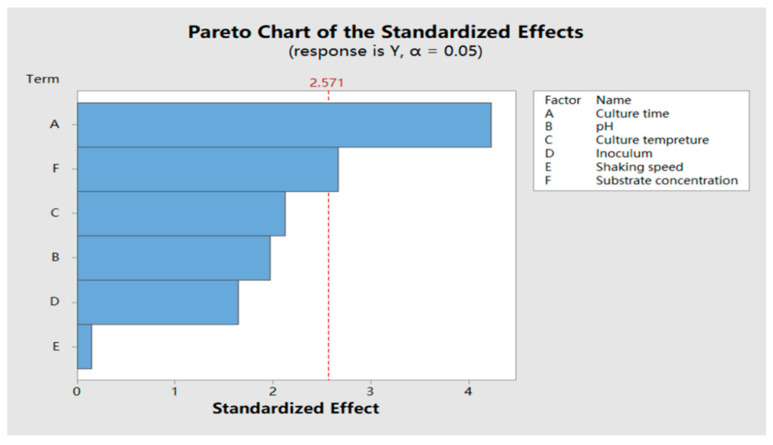
Pareto chart of standardized factors (α = 0.05).

As shown in Table 3, the model is significant (*p* = 0.034 < 0.05). As shown in the Pareto chart of standardized factors (Figure 5), when the confidence interval was set as 95%, culture time showed the most significant effect among 6 tested factors on the resveratrol transformation rate followed by substrate concentration. Therefore, two factors with significance were chosen for a response surface optimization test.

#### 2.5.2. Central Composite Design Test

As shown in Table 4, the effect of this model is very significant (*p* < 0.0001) and the co-relationship between dependent variables and independent variables is significant (R^2^ = 0.9870). the coefficient of adjusted determination for this model (R^2^_Adj_) was 0.9777, which indicated that our model could simulate 97.77% of response value change. Mismatch error was not significant (*p* > 0.05), which indicated, that the quadratic regression equation could analyze and predicate the transforming rate (Y). Besides, *p* values of A, B, AB, A^2^ and B^2^ were all less than 0.01, which suggested their significant effects on transformation. Overall, based on our results, the model constructed is reasonable.

Design-Expert version 8.0.6 was used for the optimization, and concluded that the transforming rate could reach 92.654% after being cultured for 7.5 h when fed with 0.47 mg/mL polydatin, with other parameters set as normal level. To validate the predicted result, the experiment was replicated for three parallel experiments. The results are shown in Table 5. Our results proved that determined results were very similar with predicted transforming rate, which demonstrated the satisfying predictability.

Taken together, the optimized biotransformation condition from polydatin to resveratrol by X1 was confirmed, when fed 0.47 mg/mL polydatin in the X1 culture by 5% inoculum at 35 °C in a shaking incubator as 150 rpm and media pH was adjusted to 7.0, 92.61% polydatin was transformed into resveratrol in 7.5 h.

### 2.6. Biotransformation Using Crude Extract as Substrate

**Figure 6:**
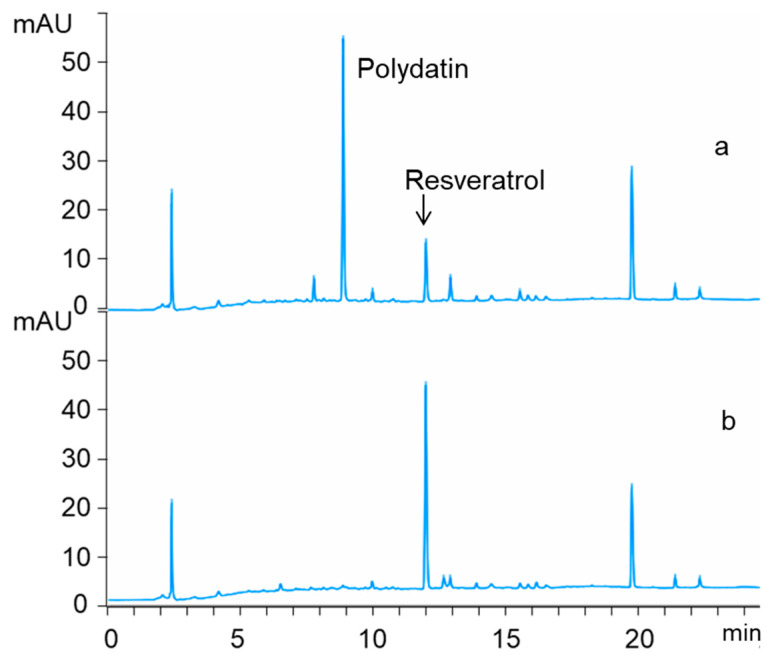
Representative HPLC chromatogram of 1 mg/mL *R. japonica* root and rhizome (RJE) before (**a**) and after (**b**) transformation by X1.

**Figure 7:**
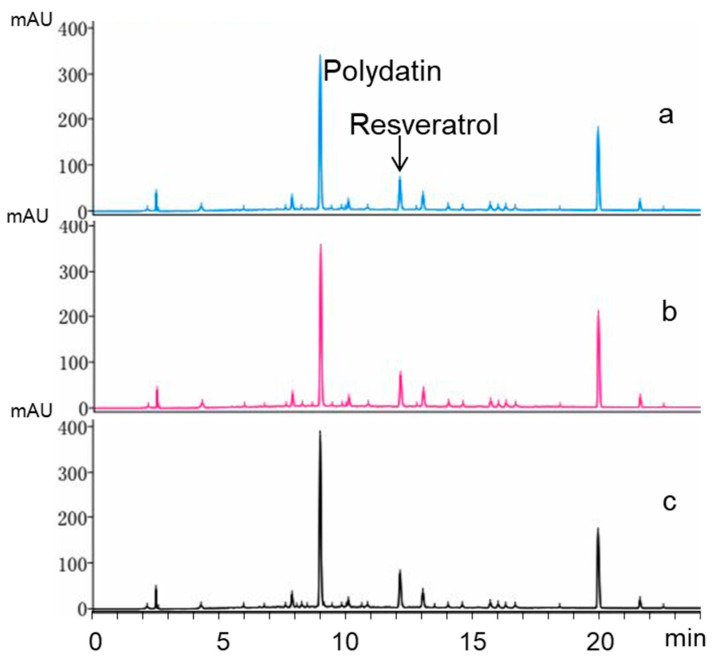
Representative HPLC chromatogram of X1 culture transforming polydatin in crude extract into resveratrol. (**a**): RJE (10 mg/mL); (**b)**: RJE (10 mg/mL) + 5% seed culture; (**c**): RJE (10 mg/mL) + X1 culture that grows to a logarithmic stage.

As mentioned above, a *Bacillus* strain was isolated and screened for transforming polydatin into resveratrol with high activity among isolated microbes. However, it would be more convenient and attractive to use crude extract of *R. japonica* Houtt (RJE) instead of pure polydatin as substrate to produce resveratrol. When fed with 1.0 mg/mL crude extract in the culture, the transforming rate of polydatin reached 92.14% (Figure 6). Unfortunately, as shown in Figure 7, after the addition of crude extract at final concentrations of 10 mg/mL at both initial and logarithmic stages, transformation from polydatin to resveratrol were significantly inhibited. Our results also indicated that the inhibition of the biotransformation process was not due to the growth inhibition. In the published results using crude extract of *R. japonica* as substrate for the production of resveratrol, the concentrations of extract are usually also very limited [19,20,21,22,23]. There must be something in the extract responsible for the biotransformation inhibition of β-d-glucosidase, which is still not clear.

Based on the published results, the hydrolysis activity of β-d-glucosidase can be strongly feedback inhibited by its product, β-d-glucose, due to the bond of glucose to the active site of glucosidase and, therefore, the real substrate cannot bind to the active site [30]. To find out the key ingredients affecting the biotransformation of polydatin by X1 strain in RJE, the content of free glucose was determined using HPLC-refractive index detector (RID), and our results showed that there was only a trace amount of glucose in the RJE (data not shown). We further hypothesized that there might be oligosaccharides in the RJE, which was hydrolyzed to glucose and affected the biotransformation. The RJE was dissolved in pure ethanol and the solution was concentrated and added into the X1 culture with equivalent concentration of 10 mg/mL RJE. Our results showed that the transformation was still strongly inhibited. Furthermore, it was reported that emodin, the main anthoraquinone compound, showed significant α-glucosidase inhibition activity. An experiment using pure emodin standard with polydatin in X1 culture was conducted, and our results indicated no transformation inhibition effects. To find out the compounds with β-glucosidase inhibition activity, RJE was substrated to partition subsequently using petroleum ether, dichloromethane, ethyl acetate and n-butanol. Each partition fraction was concentrated to dryness and added into the X1 culture with polydatin. Our results demonstrated that ethyl acetate fraction showed the most significant inhibition activity (Table 6). Consistent with our result, it was reported that vanicoside A and B from the Japanese knotweed of same genus, *Polygonum sachalinense* showed β-d-glucosidase inhibition activity with IC_50_ as 59.9 and 50.5 μM, respectively [31]. Plants with a close relationship usually contain similar compounds. This reported result partially explained why the biotransformation was inhibited when using RJE as substrate. However, due to the lack of a standard compound, the contents of vanicoside A and B in RJE were not determined. Considering the structures of vanicoside A and B, the hydroxyl groups of sucrose were substituted with several phenylpropanoyl moieties with an ester bond but not a glucosidic bond. Therefore, the glucose moiety can enter the active site of glucosidase but cannot be hydrolyzed, and act as a β-d-glucosidase inhibitor.

## 3. Discussion

There are several groups working on the production of resveratrol using microbe biotransformation, especially using fungi strains. It has been reported, that *Rhizopus microspores* hydrolyzed polydatin to resveratrol and, after 36 h, the concentration of resveratrol increased from 0.04 g/L to 0.34 g/L [19]. A β-d-glucosidase purified from *Aspergillus niger* SK 34.002 increased the resveratrol content in crude extract of *R. japonica* by 6.9 times after 4 h hydrolysis [20]. *B. shaffordii* (CGMCC 13129) was reported to transform 93.1% polydatin to resveratrol after fermentation at 37 °C for 8 h [23]. However, there are very few reports using crude extract from *R. japonica* as substrate instead of purified polydatin in microorganism biotransformation and the concentrations of crude extract are all very limited. In this study, we tried to use the RJE as substrate and determined its transformation rate using the X1 strain and the transformation conditions were optimized using the response surface method. However, the transformation can be inhibited strongly when the concentration of RJE was 10.0 mg/mL. It was reported that, two compounds, vanicoside A and B isolated from *Polygonum sachalinense* showed significant β-d -glucosidase activity inhibition [32]. Vanicoside A and B were purified from the ethyl acetate fraction of the *P. sachalinense* methanol extract. We tested the inhibition activity of ethyl acetate fraction of RJE and found it was the only fraction inhibited the transformation from polydatin to resveratrol. Furthermore, the polydatin also can be enriched in the same fraction during the partition process. Overall, more research is needed for the effective biotransformation of polydatin to resveratrol using RJE as substrate.

## 4. Materials and Methods

### 4.1. Plant Sample

A whole fresh plant was collected in August 2018 in the medicinal herb garden of School of Traditional Chinese Materia Medica, Shenyang Pharmaceutical University, and was verified by associate professor Jia Lingyun as *Reynoutria japonica* Houtt. The plant was divided into several parts: leaves, stems, root phloem, root xylem and part of the tissues were dried at 60 °C until constant weight. The dried materials were grounded into fine powder and sealed in tube for content determination of polydatin, resveratrol and emodin using a Hitachi HPLC (Tokyo, Japan) system coupled with DAD detector (L-2420), auto-sampler (L-2200), column oven (AT-330), and reverse phase column (Waters, Milford, MA, USA, XSELECT™ HSS C_18_, 4.6 × 250 mm, 5 µm).

Besides, plant material used for extraction was purchased from Guoda TCM pharmacy (Shanghai, China) and authorized by associate professor Jia Lingyun, as dried root and rhizome tissue of *Reynoutria japonica* Houtt. The plant materials were powdered to pass through 120 meshes and extracted to obtain RJE as described in Section 4.8.

### 4.2. Chemical Reagents

Standard compounds of polydatin and resveratrol were purchased from Meilun Biotech Ltd. (Dalian, China) sodium hypochlorite, sodium hydroxide, hydrochloric acid, sodium chloride, algae agar, yeast extract, peptone, glucose, ethylenediamine tetraacetic acid tetrasodium salt (Na_2_EDTA), Tris of analytical grade were purchased from Fuyu Fine Chemical Ltd. (Tianjin, China). Potato was purchased in a local market. HPLC grade methanol and acetonitrile were purchased from Yuwang Chemical Ltd. (Shandong, China). HPLC grade water were purchased from Wahaha Group Ltd. (Tianjin, China). Ginwiz Biotech Ltd. (Tianjin, China) provided the service of primer synthesis and polymerase chain reaction (PCR) product sequencing. The 2 × Taq master premix, DNA ladder, Goldview, and gel elution kit were purchased from Com Win Biotech Co., Ltd. (Beijing, China)

### 4.3. Isolation of Endophytes from Root Tissue of R. japonica

The root tissue was flushed under tap water to remove dust and dirt, cut into fragments of 5 cm, and rinsed in 75% ethanol for 1 min, followed by sterilization in 2% NaClO solution for 10 min, and finally washed with sterile distilled water 5 times. The sterile plant tissue was cut into small pieces and homogenized in a glass homogenizer with a small amount of sterile distilled water. Homogenized plant tissues were diluted with sterile water to the final concentrations of 10^−1^, 10^−2^, 10^−3^, 10^−4^ and 10^−5^ and 200 μL dilutions were plated on Potato Dextrose Agar (PDA) and Lysogeny Broth (LB) media followed by incubation at 28 °C and 37 °C, respectively. Wash water in the last step of sterilization was simultaneously plated on the LB and PDA media as a control.

### 4.4. Screening of Transforming Polydatin to Resveratrol

Isolated microbes were cultured and 5% seed culture was inoculated in liquid media containing 0.125 mg/mL polydatin and cultured at suitable temperatures at 150 rpm in a shaking incubator for 48 h; 1.5 mL 95% ethanol was added into 0.4 mL of each culture, mixed and sonicated for 10 min, followed by centrifuging at 12,000 rpm, for 10 min. The supernatant was further filtered through 0.22 μm microfiltration membrane and 10 μL was applied for HPLC determination.

### 4.5. Content Determination of Polydatin, Resveratrol and Emodin in Different Plant Tissues and in X1 Culture

HPLC detection was conducted using an Agilent HPLC instrument coupled with Eclipse XDB-C_18_ (4.6 × 150 mm 5 μm) at 306 nm at 30 °C. HPLC grade acetonitrile and water containing 0.1% formic acid were used as mobile phase A and B. The gradient elution condition was shown in Table 7. Standard curves were prepared using concentration (C) and peak area (A) of resveratrol and polydatin standard compound, respectively. The transformation rate was calculated using the equation [25]: polydatin: A = 42895C − 234.38 (R² = 0.9999, linear range: 0.010–0.800 mg/mL); resveratrol: A = 56488C + 152.94, (R² = 0.9998, linear range: 0.005–0.400 mg/mL); emodin: A = 54984C + 227.71, R² = 0.9999, (linear range: 0.001 mg/mL–0.400 mg/mL).

Dried powder of different plant tissues was weighed accurately to about 50 mg in an Eppendorf tube and 1.5 mL methanol was added. The mixture was sonicated for 30 min and followed by centrifugation at 12,000 rpm for 10 min. The supernatant was withdrawn and filtered using a 0.45 μM filter; 10 μL was used for HPLC analysis.

Transformation rate (%) = (increased resveratrol concentration/original polydatin concentration) × (390.40/228.25) × 100, in which 228.25 and 390.40 are the molecular weight of resveratrol and polydatin, respectively.

### 4.6. 16S rRNA Amplification, Sequencing and Phylogenetic Tree Construction of Screened Microbe

The X1 was cultured in liquid media and the cell pellet was collected by the centrifuging of bacterial culture and the genomic DNA was extracted following the standard method reported by Sambrook [33] and used as template. Universal primers 27F and 1492R were used to amplify the 16S rRNA [34]. The PCR profile comprised initial denaturation at 94 °C for 5 min, followed by 30 amplification cycles of denaturation at 94 °C for 30 s, annealing at 54 °C for 40 s and extension at 72 °C for 90 s, and a final extension at 72 °C for 10 min. The PCR product was determined using agarose gel electrophoresis together with a DNA ladder. The amplicon was sequenced thoroughly using 27F, and 1492R. The sequences were proofread, spliced and a Blast n analysis was conducted in EzBiocloud (https://www.ezbiocloud.net/identify) website [35]. The phylogenetic tree was constructed using the neighbor-joining algorithm in MEGA 7 [36].

### 4.7. Identification of Biotransformation Product

Samples obtained from 4.4 were analyzed using LC-MS with methanol: water: formic acid (40:59.94:0.06) as mobile phase. A YMC-Pack ODA-A (5 μm, 4.6 × 250 mm) was used in the compound separation monitored at 306 nm at 35 °C and positive ion scanning. Furthermore, the biotransformation product of the same retention time and ultraviolet (UV) spectrum with resveratrol standard was purified using a preparative HPLC. The purified product was dried completely and dissolved in DMSO-*d*6 and analyzed using ^1^H-NMR (600 MHz). Our data demonstrated the identical chemical shift and coupling constant with published data of resveratrol [30].

### 4.8. Optimization of Biotransformation Condition

The effects of six parameters including culture time, initial pH, inoculation rate, culture temperature, shaking speed and substrate concentration were investigated using the Plackett–Burman (PB) test [37]. In the test, n was set as 12, every factor was set as two levels. Minitab 17 (Minitab Inc, State College, PA, USA) software was applied to evaluate the effects and significance of each factor, in order to confirm the significance factor. Factor levels of the PB test design are shown in Table 8.

As shown above, significant factors (A: culture time and B: substrate concentration) from the PB test were chosen for the central composite design test [38]. Five levels were set (−α,−1,0,1,α) for two factors as shown in Table 9. In total, 13 tests were run in this experiment and the results were analyzed using Design-Expert version 8.0.6 software (Stat-Ease, Inc., Minneapolis, MN, USA) in order to obtain the optimal parameter level for resveratrol yield (Y). Other parameters were set as per initial pH value as 7.0, 5% inoculation rate at 35 °C and 150 rpm.

### 4.9. Biotransformation Using Crude Extract as Substrate

We extracted 1 kg powder two times with 8 L 70% ethanol using the reflux method for two hours twice. The solvent was recycled under reduced pressure and further freeze dried to obtain the dried crude extract. The extract was then weighted and autoclaved at 121 °C for 20 min. Crude extract was dissolved in autoclaved LB media to a final concentration of 10 mg/mL at initial inoculation. The other parameters were set as optimal as concluded in Section 4.7.

### 4.10. Inhibition Effects of Glucose on the Biotransformation from Polydatin to Resveratrol

Different amounts of glucose were added in 50 mL LB media containing 0.47 mg/mL polydatin and 5% seed culture in 150 mL Erlenmeyer flasks to final concentrations of 0.00, 0.125, 0.250, 0.50, 1.00 and 2.0 mg/mL glucose. Other fermentation conditions were set according to the optimized conditions. The biotransformation rate of resveratrol in each group was determined using HPLC and calculated as described in Section 4.4.

## 5. Conclusions

In this study, 5 fungi and 6 bacteria were isolated as endophytes from *R. japonica* rhizome phloem tissue and 5 of them were proved to transform polydatin to resveratrol using HPLC screening. Among the five microbes, a *Bacillus* strain achieved the highest transformation rate and was characterized as *B. aryabhattai* by phylogenetic tree analysis using its 16S rRNA sequence. LC-MS and ^1^H-NMR were used to confirm the final product as trans-resveratrol. The fermentation condition was optimized using the response surface method. We also found that the reaction was strongly inhibited when 10.0 mg/mL RJE was used as substrate. Several experiments were carried out and we found only the ethyl acetate fraction showed inhibitory activity. Our results provided an alternative method for the production of resveratrol using a crude extract of *R. japonica* as substrate in an environment-friendly manner. However, the coexistence of polydatin and inhibitory compounds in ethyl acetate make the biotransformation using RJE as substrate difficult to scale up.

## Figures and Tables

**Table 1:** Contents determination of polydatin, resveratrol and emodin in different tissues of *R.japonica*.

Tissues Tested	Polydatin (mg/g)	Resveratrol (mg/g)	Emodin (mg/g)
Leaf	N.D.	<0.2	N.D.
Stem	<0.2	<0.02	N.D.
Rhizome-phloem	47.57 ± 2.21	3.81 ± 0.95	0.80 ± 0.16
Rhizome-xylem	14.77 ± 1.04	0.64 ± 0.36	0.21 ± 0.11
Root-phloem	33.20 ± 2.95	2.13 ± 0.25	0.85 ± 0.31
Root-xylem	11.47 ± 1.36	0.57 ± 0.15	0.32 ± 0.17

Note: N.D.: none detected under current analysis condition.

**Table 2:** Biotransformation rate from polydatin to resveratrol of positive endophytes.

No.	Biotransformation Rate (%)	Organism	Accession Number
Z1	37.13 ± 2.16	*Penicillium oxalicum*	MT795727
Z3	47.77 ± 1.68	*Penicillium steckii*	MT795729
Z4	25.88 ± 3.24	*Cladosporium cladosporioides*	MT795730
Z5	26.74 ± 1.95	*Penicillium brasilianum*	MT795731
X1	97.44 ± 2.57	*Bacillus aryabhattai*	MT792074

**Table 3:** Variance analysis of Plackett–Burman (PB) test.

Factors	*F* Value	*p* Value
Models	6.02	0.034
Culture time (h)	17.89	0.008
Initial medium pH	3.87	0.106
Inoculation rate (%)	2.70	0.161
Culture temperature (°C)	4.53	0.087
Shaking speed (rpm)	0.02	0.889
Substrate concentration (mg/mL)	7.10	0.045

**Table 4:** Variance analysis of regression model.

	Quadratic Sum	Degree of Freedom	Mean Square	*F* Value	*p* Value
Model	7866.48	5	1753.30	106.40	<0.0001
A	728.43	1	728.43	49.26	0.0002
B	714.90	1	714.90	48.35	0.0002
AB	1368.11	1	1368.11	92.53	<0.0001
A^2^	1625.33	1	1625.33	109.92	<0.0001
	4009.66	1	4009.66	271.17	<0.0001
Residual error	103.50	7	14.79		
Mismatch error	61.10	3	20.37	1.92	0.2677
Pure error	42.40	B^2^4	10.60		
Sum	7969.99	12			
R^2^ = 0.9870, R^2^_Adj_ = 0.9777					

**Table 5:** Transforming rates of three parallel experiment and statistic analysis.

No.	Biotransformation Rate Y (%)	Average (%)	Relative Standard Deviasion (RSD) (%)
1	93.33	92.61	0.73
2	92.53		
3	91.98		

**Table 6:** Effects addition of different components on the biotransformation of polydatin to resveratrol.

Components Tested	X1 Culture and Polydatin Addition	Polydatin Transformation Rate (%)
RJE 1.0 mg/mL	Initial stage 5%	92.15 ± 2.51
RJE 5.0 mg/mL	Initial stage 5%	20.58 ± 4.23
RJE 10.0 mg/mL	Initial stage 5%	<5%
Glucose 2.0 mg/mL	Initial stage 5% + 0.47 mg/mL polydatin	<5%
ethanol soluble fraction equivalents to RJE 10.0 mg/mL	Initial stage 5%	<5%
emodin equivalents to RJE 10.0 mg/mL	Initial stage 5% + 0.47 mg/mL polydatin	94.43 ± 3.06
petroleum fraction equivalents to RJE 10.0 mg/mL	Initial stage 5% + 0.47 mg/mL polydatin	95.86 ± 4.25
dichloroform fraction equivalents to RJE 10.0 mg/mL	Initial stage 5% + 0.47 mg/mL polydatin	86.43 ± 1.56
ethyl acetate fraction equivalent to RJE 10.0 mg/mL	Initial stage 5% + 0.47 mg/mL polydatin	<5%
n-butanol fraction equivalents to RJE 10.0 mg/mL	Initial stage 5% + 0.47 mg/mL polydatin	91.75 ± 5.91
water fraction equivalents to RJE 10.0 mg/mL	Initial stage 5% + 0.47 mg/mL polydatin	98.25 ± 4.77

**Table 7:** Gradient HPLC elution condition.

t/min	A/%	B/%
0	5	95
3	15	85
10	30	70
20	50	50
25	100	0

**Table 8:** Parameters and levels setting in PB test.

Factor	Level	
	−1	1
Culture time (h)	6	10
Initial medium pH	6.5	7.0
Inoculation rate (%)	3	7
Culture temperature (℃)	33	37
Shaking speed (rpm)	120	180
Substrate concentration (mg/mL)	0.35	0.7

**Table 9:** Parameters and level setting in central composite design.

Factors	Levels				
	−α	−1	0	1	α
A-culture time/h	5.17	6.00	8.00	10.00	10.83
B-substrate concentration/(mg/mL)	0.24	0.30	0.45	0.60	0.66
